# rBahadur: efficient simulation of structured high-dimensional genotype data with applications to assortative mating

**DOI:** 10.1186/s12859-023-05442-6

**Published:** 2023-08-18

**Authors:** Richard Border, Osman Asif Malik

**Affiliations:** 1 Neurology and Computer Science, University of California, Los Angeles, 675 Charles E Young Dr S, Los Angeles, CA 90095 USA; 2 Applied Mathematics and Computational Research Division, Lawrence Berkeley National Laboratory, 1 Cyclotron Road, Berkeley, CA 94720 USA

**Keywords:** Assortative mating, Multivariate Bernoulli, Genotype simulation

## Abstract

Existing methods for generating synthetic genotype data are ill-suited for replicating the effects of assortative mating (AM). We propose rb_dplr, a novel and computationally efficient algorithm for generating high-dimensional binary random variates that effectively recapitulates AM-induced genetic architectures using the Bahadur order-2 approximation of the multivariate Bernoulli distribution. The rBahadur R library is available through the Comprehensive R Archive Network at https://CRAN.R-project.org/package=rBahadur.

**Supplementary Information:**

The online version contains supplementary material available at 10.1186/s12859-023-05442-6.

## Background

The simulation of realistic genotype/phenotype data is a fundamental tool in statistical genetics and is essential for the development of robust statistical methods for the analysis of genome-wide data. As such, much prior effort has focused on generating synthetic data that recapitulate salient characteristics of genetic marker data, including local linkage disequilibrium (LD) structure induced by variable recombination rates [1–3], relationships between local LD structure and allelic effects [4], and the many consequences of drift, admixture, and geographic stratification [5, 6].

Despite these advances, existing methods are ill-suited for generating synthetic genotype/phenotype data reflecting the consequences of recent assortative mating (AM); in contrast to the effects of recombination, which yields banded covariance structures, AM induces dense covariance structures reflecting sign-consistent dependence among all causal variants across the genome [7, 8]. On the other hand, there is substantial recent evidence that AM is widespread [8–10] and complicates the interpretation of many commonly applied methods in statistical genetics, including heritability estimation [11], genetic correlation estimation [8, 12], and Mendelian randomization [13]. Efficient simulation methods for generating high-dimensional genotype/phenotype data congruent with the consequences of AM will be critical to the development of robust analytic tools.

**Fig. 1:**
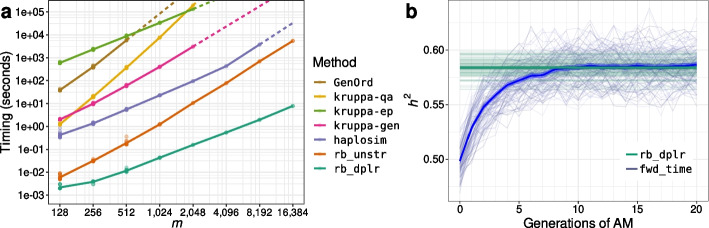
**a** Cross-method comparison of single-threaded wall time for generating *m*/4 samples from the *m*-dimensional MVB distribution on a log-log scale. The proposed rb_dplr algorithm scales linearly in sample size and problem dimension. Both rb_dplr and the unstructured variant rb_unstr outperform existing methods including haplosim [3], three methods implemented by Kruppa et al. (kruppa-*) [1], and GenOrd [15] Solid lines reflect linear splines with fixed knots fitted to numerical experiment results and dashed lines reflect extrapolations. **b** Drawing genotypes directly from their equilibrium distribution under AM. Comparison of heritabilities in synthetic genotype/phenotype data generated using rb_dplr to sample from the appropriate MVB distribution, versus the forward-time approach of Border et al. [8]. Best-fit lines and standard-errors summarize variation across 100 replicates with 2000 haploid causal variants for 8000 individuals, for phenotypes with panmictic heritability cross-mate phenotypic correlation both fixed to 0.5

SNP haplotypes subject to AM-induced long-range dependencies can be represented mathematically by the *m*-dimensional multivariate Bernoulli (MVB) distribution [14], which is challenging to sample from; existing methods require $O(m^2)$ or more operations to draw a vector of *m* haplotypes [1, 3, 15, 16] (see Fig. 1), which becomes infeasible in high dimensions. As such, existing methods for synthesizing AM-consistent marker data at scale obviate this problem by generating genotype / phenotype data assuming random mating and subsequently proceeding through multiple generations of forward-time simulation, complete with mating and meioses [2, 8, 11]. However, these methods require repeatedly shuffling the elements of large arrays and simulating the genotypes of a large number of individuals to obtain a relatively small sample of unrelated individuals, making them cumbersome in the context of methods development.

In the current manuscript, we introduce a novel collection of efficient methods for directly sampling high-dimensional MVB random variables satisfying particular moment conditions (i.e., admitting a Bahadur order-2 representation; [17]). In particular, we propose the rb_dplr algorithm, which exploits the diagonal-plus-low-rank correlation structure induced by AM to generate MVB samples using only *O*(*m*) operations. We then present numerical experiments demonstrating that the proposed methods outperform existing direct sampling methods and verify that they faithfully represent the effects of AM by comparing results to forward-time simulations. We provide these methods, together with a collection of utilities for characterizing the equilibrium distribution of haplotypes under AM, in rBahadur, an open-source library for the R programming language.

## Implementation

### Overview of the rBahadur library

The rBahadur library consists of three component collections: First, we provide two general-purpose MVB samplers, rb_unstr and rb_dplr, the implementation of which we discuss in the following section. Second, we provide utilities for modeling equilibrium AM, including a set of convenience functions for computing equilibrium parameters given initial conditions and for parametrizing the corresponding MVB distribution. Third, we provide a routine for end-to-end simulation that combines the first two components to efficiently construct equilibrium genotypes and phenotypes given population parameters.

### Bahadur approach to the MVB distribution

Suppose $X_1, \ldots , X_m$ are Bernoulli random variables with means $\mu _i :=\mathbb {E}[X_i]$. When the variables are independent, the distribution of $(X_1, \ldots , X_m)$ is simply $p_{[1]}(x_1, \ldots , x_m) :=\prod _{i=1}^m \mu _i^{x_i} (1-\mu _i)^{1-x_i}$, but this is not the case in general. Bahadur [17] showed that the distribution of an MVB takes the form

1 $$\begin{aligned} p(x) = p_{[1]}(x) f(x), \end{aligned}$$

where $x = (x_1, \ldots , x_m)$. The function *f* in (1) is defined as

2 $$\begin{aligned} f(x) = 1 + \sum _{i< j} r_{ij} z_i z_j + \sum _{i<j<k} r_{ijk} z_i z_j z_k + \cdots + r_{1 \cdots m} z_1 \cdots z_m, \end{aligned}$$

where $z_i :=(x_i - \mu _i)/\sqrt{\mu _i(1-\mu _i)}$ and $r_{i_1 \ldots i_n} :=\mathbb {E}[ z_{i_1} \cdots z_{i_n} ]$. The means $(\mu _i)$ and mixed moments $(r_{ij}), (r_{ijk})$, and so forth, characterize the MVB and are comprised of $2^m-1$ parameters. This exponential dependence on *m* makes working with general MVBs challenging.

We consider Bahadur order-2 approximations to the distribution in (1); i.e., we assume that $r_{i_1 \cdots i_n} = 0$ for $n \ge 3$. Thus, the order-2 MVB distribution is fully characterized by its means $(\mu _i)$ and correlations $(r_{ij})$. rBahadur provides two methods for sampling from this distribution. The first algorithm (rb_unstr) can handle generic correlation matrices and requires $O(m^2)$ operations to sample from the *m*-dimensional MVB. The second algorithm (rb_dplr) is developed specifically for the case when the correlation matrix is diagonal-plus-low-rank (DPLR; i.e., $(r_{ij}) = \textbf{D} + \textbf{U} \textbf{U}^T$ where **D** is diagonal and **U** is $m \times c$ for some $c \ll m$) and requires *O*(*mc*) operations. Both methods sample the entries of the random vector sequentially: First, a realization of $X_1$ is drawn. Then, subsequent variables $X_n$ are drawn *conditionally* on the realization of the previously drawn variables $X_1, \ldots , X_{n-1}$ for $2 \le n \le m$. For further details, see Additional file 1.

### Equilibrium distribution of causal variants under AM

Here we demonstrate how the DPLR order-2 MVB distribution is used to model the consequences of assortment. Consider the equilibrium distribution of haploid causal variants $X_1,\dots ,X_m$ with allele frequencies $\mu _1,\dots ,\mu _m$ under primary-phenotypic assortative mating for an additive phenotype with panmictic heritability $h^2_0$, panmictic genetic variance $\sigma ^2_{g,0}=h^2_0$, and cross-mate phenotype correlation *r*. Following Nagylaki [7], the equilibrium heritability is

$$\begin{aligned} h^2_\infty = \frac{1}{2r}\left( (1-h^2_0)^{-1} - \sqrt{(1-h^2_0)^{-2} - 4 r h^2_0 (1-h^2_0)^{-1}} \right) , \end{aligned}$$

and the equilibrium cross-mate genetic correlation and genetic variance are respectively $r_{g,\infty }=r\cdot h^2_\infty$ and $\sigma ^2_{g,\infty } = \sigma ^2_{g,0}/(1-r_{g,\infty })$. Additionally, we denote the equilibrium phenotypic variance $\sigma ^2_{y,\infty }$ and the standardized haploid effects $\varvec{\beta }$.

Assuming casual variants are unlinked at panmixis, the correlation matrix of causal haploid variants will be of the form $\textbf{R}=\textbf{D}+{\varvec{\phi} }{\varvec{\phi} }^T$ where, following Border et al. [11], ${\varvec{\phi} }:\mathbb {R}^m\rightarrow \mathbb {R}^m$ is a function of the standardized haploid effects $\varvec{\beta }$ given elementwise by

3 $$\begin{aligned} \phi _k = \sqrt{\sigma ^2_{y,\infty }\mu _k(1-\mu _k)/(8\beta _k^2r)} \left( \sqrt{4\beta _k^2 r / \sigma ^2_{y,\infty } +(1-r_{g,\infty })^2} -(1-r_{g,\infty }) \right) , \end{aligned}$$

and $\textbf{D}$ is the diagonal matrix with entries $\textbf{D}_{kk}=1-\phi _k^2$. Setting $\textbf{U}={\varvec{\phi}}$ allows drawing haploid causal variants from the equilibrium distribution under AM via ${\texttt {rb}\_\texttt {dplr}}$.

The ${\texttt {rBahadur}}$ library includes functions for computing equilibrium parameters under this model: vg_eq, h2_eq, and rg_eq compute equilibrium parameters given the initial conditions $\sigma ^2_{g,0}$, $h^2_0$, and *r*. Finally, am_covariance_structure parametrizes the corresponding DPLR MVB distribution for a specified set of allele frequencies, causal effects, and initial conditions, by using (3) to compute ${\varvec{\phi} }$.

### Simulating genotype/phenotype data with rBahadur

rBahadur provides the am_simulate routine for simplified end-to-end simulation of genotype/phenotype data. am_simulate requires the user to specify the panmictic heritability and mating correlation parameters, as well as the desired number of diploid causal variants and number of simulation replicates (i.e., individuals). am_simulate returns genotypes, phenotypes, as well as additional architectural components, including the allele frequencies, allele-substitution effects, and the heritable component of the generated phenotype. We provide a vignette illustrating usage of am_simulate in further detail in Additional file 1.

### Numerical experiments

Figure 1a compares the time required to generate *m* binary haplotypes for $n=m/4$ individuals under the equilibrium AM model, with $h_0^2=r=0.5$, across existing and proposed methods. The proposed rb_dplr algorithm scales linearly in sample size and problem dimension. Both rb_dplr and the unstructured variant rb_unstr outperform existing methods including haplosim [3], three methods implemented by Kruppa et al. (kruppa-*) [1], and GenOrd [15]. Results for the mipfp library [16], which had exponential time complexity, are omitted.

Figure 1b compares heritabilities associated with genotype / phenotype data as generated with rb_dplr under the equilibrium AM model versus those achieved after of up to 20 generations of the corresponding forward-time procedure, as implemented by Border et al. [8], across 100 replicates. Results were consistent across methods (comparing rb_dplr $h^2$ to mean $h^2$ values across forward-time generations 16-20, Welch’s $t(99)=0.84$, $p=0.40$). Best-fit lines and standard-errors summarize variation across 100 replicates with 2000 haploid causal variants for 8000 individuals. Here, rb_dplr provides a direct and efficient alternative to forward time approaches that can be readily incorporated into sensitivity analysis and methods development pipelines.

## Conclusions

Given that AM is both widespread [10, 11] and consequential for the interpretation of marker-based estimators [8, 11, 13], it is crucial that statistical geneticists are able to perturb random-mating assumptions when developing and evaluating methods. To this end, we have developed the rBahadur library to efficiently sample haploid causal variants under AM-induced genetic architectures. The software is open-source and freely available through Comprehensive R Archive Network at https://CRAN.R-project.org/package=rBahadur.

Our approach is limited by the requirement that the target distribution admits a second-order Bahadur approximation (i.e., there is a valid probability distribution with the specified allele frequencies and correlations). For far apart causal variants, this is of little consequence as the true values of moments up to order four are expected to be smaller than their sampling variances unless $n\gg m$, which is rarely the case in practice [11]. However, this limits applications to complex correlation structures involving both strong local LD and AM-induced global dependence. We address this by ensuring the simulation functions in rBahadur fail transparently in such cases and provide a vignette demonstrating how rBahadur can be used in conjunction with reference haplotypes to overcome this limitation in Additional file 1.

### Supplementary Information

**Additional file 1:** Supplementary note.

## Data Availability

Project name: rBahadur Project homepage: https://CRAN.R-project.org/package=rBahadur Operating system: Platform independent Programming language: R Other requirements: Not applicable License: GNU General Public License v3.0 Any restrictions to use by non-academics: None.

## Abbreviations

AM: Assortative mating
DPLR: Diagonal-plus-low-rank
LD: Linkage disequilibrium
MVB: Multivariate Bernoulli (distribution)
